# Association of Bone Mass, Falls, and Vertebral Fractures in Older Patients on Hemodialysis: The Role of Comprehensive Geriatric Assessment

**DOI:** 10.1007/s11606-026-10398-3

**Published:** 2026-05-06

**Authors:** Suzan Abou-Raya, Mohamed Mehanna, Yasser Allam, Khaled Matrawy, Shimaa ElSaied, Anna Abou-Raya

**Affiliations:** 1 Geriatric Division, Internal Medicine Department, Alexandria University Faculty of Medicine, Alexandria, Egypt; 2 Orthopedic Surgery and Traumatology Department, Alexandria University Faculty of Medicine, Alexandria, Egypt; 3 Radio-Diagnosis and Intervention, Department of Radiology, Medical Research Institute, Alexandria University, Alexandria, Egypt; 4 Nephrology Division, Ministry of Health Hospital, Alexandria, Egypt; 5 Rheumatology & Clinical Immunology Division, Internal Medicine Department, Alexandria University Faculty of Medicine, Alexandria, Egypt

**Keywords:** End-stage renal disease, Hemodialysis, Comprehensive geriatric assessment, Falls, Vertebral compression fractures, Bone mass, Osteoporosis

## Abstract

**Background:**

End-stage renal disease (ESRD) requiring hemodialysis (HD) disproportionately affects older adults, who face increased risks of falls and fractures due to age-related changes, renal osteodystrophy, comorbidities and dialysis-related factors. Although bone abnormalities and fall risk have often been examined separately in patients with ESRD, few studies have evaluated vertebral compression fractures (VCF) in older HD patients while concurrently assessing skeletal, functional and geriatric risk factors. The role of Comprehensive Geriatric Assessment (CGA) in identifying multidimensional risk factors in this context remains underexplored.

**Objective:**

To investigate associations between bone mass, falls and VCF in older adults undergoing HD, and evaluate the utility of CGA in identifying risk factors and informing care strategies.

**Participants:**

This cross-sectional study enrolled 50 older adults (≥ 65 years; mean age 67.8 (3.2), range 65–76) with ESRD on maintenance HD. Participants had multiple comorbidities, typical of this population, including anemia, cardiovascular disease and chronic liver disease.

**Main Measures:**

Bone mineral density was assessed using dual-energy X-ray absorptiometry. Falls, VCF, comorbidities, and medication use were documented via interviews and medical records. CGA included assessments of activities of daily living (ADLs), mobility, cognition, depression, nutrition and social support. FRAX scores and biochemical markers (parathyroid hormone [PTH], bone-specific alkaline phosphatase [ALP]) were analyzed.

**Results:**

Falls occurred in 28% of participants, significantly associated with prior fall history (*p* < 0.001), advanced age (*p* = 0.011), anemia (*p* = 0.040), chronic liver disease (*p* = 0.044), reduced mobility (*p* = 0.037), depression (*p* = 0.01), impaired ADLs (*p* = 0.037) and poor nutrition (*p* = 0.032). VCF were present in 14%, associated with earlier menopausal age, back pain, elevated ALP and higher FRAX scores. Osteoporosis was most prevalent at the distal radius (46%). Hip T-scores were lower in the VCF group (*p* = 0.003). PTH and ALP were significantly higher in VCF patients (*p* = 0.003), indicating impaired bone turnover.

**Conclusion:**

Older adults on HD face a substantial burden of falls and vertebral fractures due to overlapping functional, metabolic and psychosocial factors. CGA effectively identified multidimensional risks, underscoring the need for integrated preventive strategies.

**Supplementary Information:**

The online version contains supplementary material available at 10.1007/s11606-026-10398-3.

## INTRODUCTION

End-stage renal disease (ESRD) is a chronic condition marked by progressive kidney function loss, necessitating hemodialysis (HD) as a life-sustaining treatment^1^. Older patients with ESRD undergoing HD face heightened risks of reduced bone mass, falls and vertebral compression fractures (VCF)^2,3^. This condition disproportionately affects older individuals, who are particularly vulnerable to complications such as falls and fractures due to a combination of age-related physiological changes, renal osteodystrophy and the cumulative effects of comorbidities and dialysis therapy itself^1,2^.

A prominent complication of ESRD is CKD-Mineral and Bone Disorder (CKD-MBD), which is distinct from age-related osteoporosis and significantly heightens bone fragility and fracture risk in dialysis patients^3–6^. The prevalence of bone disorders in older ESRD patients on HD is substantial, ranging from 33 to 66%^1,2^. Renal osteodystrophy, a hallmark of ESRD, disrupts bone metabolism, resulting in decreased bone mass and compromised bone quality, thus elevating fracture risk^3–5^. These skeletal abnormalities, along with secondary hyperparathyroidism, vitamin D deficiency, malnutrition, and medications commonly used in dialysis, further exacerbate bone fragility^5^. Falls and fractures are significant contributors to morbidity and mortality in this population, imposing a burden on healthcare systems^6^.

Diagnosing bone loss in this population involves comprehensive assessments, including bone mineral density (BMD) evaluations using dual-energy X-ray absorptiometry (DXA) scans and analysis of biochemical markers like bone-specific alkaline phosphatase (BAP) and C-terminal telopeptide of type I collagen (CTX)^7^. Additionally, Comprehensive Geriatric Assessment (CGA) is pivotal in evaluating physical, mental and functional abilities^8^. CGA is a multidimensional, interdisciplinary diagnostic process that evaluates the medical, functional, cognitive, psychological, and social capabilities of older adults. CGA is particularly valuable for detecting and managing complex, multifactorial issues that may not be apparent in standard assessments, especially geriatric syndromes. Geriatric syndromes refer to a group of multifactorial health conditions commonly seen in older adults that do not fit into discrete disease categories but significantly impact quality of life and clinical outcomes. These include impaired mobility, cognitive decline, depression, malnutrition, falls, frailty, incontinence and functional dependence.

For older ESRD patients, CGA offers a valuable framework for identifying and managing risk factors for falls and fractures, with the potential to improve health outcomes significantly.

Despite the well-documented risk factors, limited research has explored the interrelationship between bone mass, falls, and VCF in older patients on HD. Additionally, the potential of CGA to address these risks remains underexplored. This study aimed to investigate the association between bone mass, falls, and VCF in older patients on HD. A second aim of the study was to explore the role of CGA in identifying functional, nutritional and psychological risk factors associated with falls and VCF risk in older adults undergoing HD.

## METHODS

### Study Population

This cross-sectional study included 50 adults aged ≥ 65 years with end-stage renal disease (ESRD) undergoing maintenance hemodialysis (HD) three times weekly for at least six months to ensure clinical stability and minimize early dialysis-related variability in bone metabolism and functional status. Participants were recruited from the dialysis unit and spine outpatient clinics of the Main University Hospital over a six-month period. Exclusion criteria included: pathological fractures; current use of bisphosphonates, calcitonin or hormone replacement therapy; severe life-threatening conditions; cognitive impairment precluding informed consent; and bone disorders unrelated to ESRD. The sample size was calculated to achieve 80% power at a 0.05 significance level. Written informed consent was obtained from all participants, and ethical approval was granted by the institutional ethics committee.

### Data Collection

Demographic and clinical data were collected through questionnaires and medical records. Variables included age, sex, medication use, fall history, family history of osteoporosis, back pain, sensory impairments, lifestyle factors (including smoking), menopausal age (for women), and comorbidities. ESRD-specific data included etiology, duration of disease, HD duration, and dialysis parameters from six consecutive sessions (pre/post-dialysis blood pressure, ultrafiltration volume, nadir blood pressure, and Kt/V- a standard measure of dialysis adequacy that quantifies how effectively urea is cleared from the body during a dialysis session. It represents the ratio of urea clearance (K), dialysis time (t), and the volume of distribution of urea (V)). Comorbidity burden was quantified using the Charlson Comorbidity Index (CCI)^9^. General physical examination covered cardiopulmonary, neurological, and musculoskeletal systems.

Laboratory investigations: Blood samples were analyzed for hemoglobin, corrected serum calcium (adjusted for albumin), phosphate, bone specific alkaline phosphatase (ALP), 25-hydroxy-vitamin D [25(OH)D], and parathyroid hormone (PTH).

Laboratory investigations with reference ranges and thresholds used in this study:

**Table:** 

Parameter	Measured as	Reference Range	Thresholds Used in Analysis
Hemoglobin	g/dL	12–16 (women), 13–17 (men)	Anemia defined as < 12 in women and < 13 in men
Corrected serum calcium	mg/dL (adjusted for albumin)	8.5–10.2	Hypocalcemia: < 8.5
Phosphate	mg/dL	2.5–4.5	Hyperphosphatemia: > 4.5
Bone-specific ALP	U/L	15–41	Elevated > 41
25 (OH) Vitamin D	ng/mL	30–100	Deficiency: < 20 Insufficiency: 20–29
Parathyroid Hormone (PTH)	pg/mL	15–65	Elevated: > 66

Bone mineral density (BMD) was measured using dual-energy X-ray absorptiometry (DXA) at the lumbar spine, femoral neck/total hip and distal radius. BMD values were expressed in grams per square centimeter (g/cm^2^). Osteoporosis classification followed WHO criteria: T-score between −1 and −2.5 was defined as osteopenia; T-score ≤ −2.5 as osteoporosis; and T-score ≤ −2.5 with a history of fracture as severe osteoporosis. In alignment with clinical practice, participants with fragility fractures, regardless of BMD, or those with osteopenia and a high fracture risk (based on FRAX scores) were also classified as having osteoporosis for the purposes of this study. High fracture risk is defined as a 10-year probability of ≥ 20% for major osteoporotic fracture and/or ≥ 3% for hip fracture.

### Assessment

Comprehensive Geriatric Assessment (CGA) is a multidimensional, interdisciplinary diagnostic process designed to evaluate the medical, functional, psychological and social capabilities of older adults. CGA provides a coordinated and integrated plan for treatment and long-term follow-up. It is ideally conducted by an interdisciplinary team including physicians, nurses, dietitians, physiotherapists, and social workers. CGA provides a holistic understanding of patient needs and vulnerabilities***.*** In this study, CGA was conducted by a multidisciplinary team using validated assessment tools. The CGA was led by a multidisciplinary clinical team comprised of geriatricians, a nephrologist, a rheumatologist and an orthopedic surgeon. Nursing staff and a social worker assisted with selected assessment domains, including functional status and social support under the supervision of the core clinical team.

Functional status was assessed using the Katz Activities of Daily Living (ADL) Index, with scores ranging from 0 to 12; scores ≤ 7 indicated impairment^10^. Mobility was evaluated by the Timed Up and Go (TUG) test, where times ≥ 13.5 s indicated increased fall risk^11^. Cognitive function was measured using the Mini-Mental State Examination (MMSE), with scores ranging from 0 to 30^12^. Depressive symptoms were screened using the Geriatric Depression Scale (GDS-15), with scores ≥ 5 suggesting possible depression and ≥ 10 indicating probable depression^13^. Nutritional status was assessed using the Mini Nutritional Assessment (MNA), where scores ≥ 24 indicated normal nutrition, 17–23.5^14^. The Fall Risk Assessment Tool (FRAT) was used to assess fall risk based on history, medications, sensory deficits, and mobility where scores ≥ 5 indicated high fall risk^15^. The Clinical Frailty Scale (CFS), developed by Rockwood, was used to assess frailty status which ranges from 1 (very fit) to 9 (terminally ill) and a score of ≥ 5 considered frailty^16^. A medication review was conducted to identify polypharmacy (≥ 5 medications), potentially inappropriate medications, and fall-risk-increasing drugs including sedatives, anticholinergics and antihypertensives. Social support was assessed through interviews addressing living arrangements, frequency of contact with family/friends, and availability of help with daily tasks.

Falls were assessed via patient questionnaires documenting events in the past year, including circumstances and consequences. Vertebral fractures were evaluated using lateral thoracolumbar spine X-rays, non-contrast dorsal CT scans, and FRAX scores for major osteoporotic fractures^17^^,18^.

### Outcome Measures

Primary outcomes: a) the prevalence of falls within the past 12 months, characterized by number, frequency, and contextual details (e.g., location, activity, injury sustained), as well as the prevalence of VCF confirmed by imaging; b) BMD assessed at the lumbar spine, femoral neck/total hip, and distal radius. Osteoporosis was defined as a predefined clinical outcome based on established diagnostic criteria, including a T-score ≤ − 2.5, a history of fragility fracture irrespective of BMD, or osteopenia with a high FRAX-estimated fracture risk.

Secondary outcomes: included individual geriatric domains assessed through comprehensive geriatric assessment, including reduced mobility (Timed Up and Go test), depression (GDS-15), impaired ADL (Katz Index), poor nutritional status (MNA), medication safety (medication review) and limited social support (interviews). These domains were analyzed individually in relation to clinical outcomes.

### Ethical Considerations

The study adhered to the principles of the Declaration of Helsinki. Ethical approval was obtained from the local ethics committee, and informed consent was obtained from all participants ensuring their rights, safety, and confidentiality was prioritized throughout the research.

### Statistical Analysis

Data was entered into Microsoft Access and analyzed using SPSS version 22. Descriptive statistics summarized baseline characteristics using means, standard deviations, and percentages. Correlation coefficients and regression analyses were performed to identify relationships between bone mass, falls, and VCF.

## RESULTS

### Demographic and Clinical Data

The demographic and clinical characteristics of the study participants are depicted in Table 1. The cohort consisted of 50 older patients on HD with an age range of 65–76 years (mean age of 67.82 years; 62% male; BMI: 29.02 kg/m^2^) and an average dialysis duration of 7.6 years. Eighteen percent reported smoking. Hypertension (38%) and unknown causes (30%) were the primary reasons for initiating dialysis. Notable findings included a history of falls in 32%, VCF in 14%, and prevalent back pain in 46%. Sensory impairments were common-vision problems (58%) and hearing difficulties (18%). The average CCI was 2.84, indicating a high burden of chronic illnesses, and frailty was marked (mean frailty index of 3.80). Functional impairments were prevalent with impaired ADLs (score 5.32) and mobility limitations (TUG: 17.18 s). Laboratory results showed anemia (mean hemoglobin: 9.29 g/dL), elevated bone turnover markers (mean PTH (211.74 pg/mL; mean ALP (201.04 U/L) and severe vitamin D deficiency (mean 6.21 ng/mL).

**Table 1:** Baseline Demographic and Clinical Data of the Study Participants

Characteristic	Value
Age (years), mean (SD)	67.82(3.23)
Gender (Males/Females), n (%)	31(62)/19(38)
BMI (kg/m^2^), mean (SD)	29.02(7.71)
Age of Menopause (years), mean (SD)	47.16(1.95)
Smokers/Non-smokers, n (%)	9(18)/41(82)
Duration of Dialysis (years), mean (SD)	7.57(4.76)
Cause of Dialysis, n (%)	
Hypertension	19(38)
Diabetic nephropathy	5(10)
Obstructive uropathy	7(14)
Contrast uropathy	2(4)
NSAID use	2(4)
Unknown cause	15(30)
Back pain, n (%)	23(46)
History of Falls n (%)	16 (32)
History of previous fractures n (%)	8 (16)
History of previous vertebral compression fractures	7(14)
Family history of Osteoporosis, n (%)	1(2)
Family history of Fractures, n (%)	1(2)
FRAX-Hip, mean (SD)	0.006 (0.005)
FRAX-MOF, mean (SD)	0.059 (0.016)
FRAT, mean (SD)	5.76 (2.98)
Vision problems, n (%)	29(58)
Hearing Problems, n (%)	9(18)
Charlson Comorbidity Index (CCI)	2.84(1.81)
ADL score, mean (SD)	5.32(1.55)
TUG (sec), mean (SD)	17.18 (3.43)
MNA, mean (SD)	25.93 (2.26)
MMSE, mean (SD)	26.76 (2.42)
GDS-15, mean (SD)	8.28 (2.24)
Frailty Index, mean (SD)	3.80 (1.77)
Kt/v, mean (SD)	1.31(0.30)
Ultrafiltration Volume (L), mean (SD)	2.88 (0.76)
Pre-Dialysis Systolic BP (mmHg), mean (SD)	126.98 (13.97)
Pre-Dialysis Diastolic BP (mmHg), mean (SD)	79.58 (5.42)
Dialysis Systolic BP (mmHg), mean (SD)	114.32 (10.66)
Dialysis Diastolic BP (mmHg), mean (SD)	73.81 (84.00)
Post-Dialysis Systolic BP (mmHg), mean (SD)	115.52 (11.78)
Post-Dialysis Diastolic BP (mmHg), mean (SD)	73.82 (6.87)
Comorbidities, n (%)	
Hypertension	34(68)
Ischemic heart disease	11(22)
Heart failure	1(2)
Diabetes Mellitus	10(20)
Muscle weakness	6(12)
Foot problems	18(36)
Hyperthyroidism	1(2)
Chronic liver disease	5(10)
Rheumatoid arthritis	2(4)
Polypharmacy, n (%)	100(100)
Steroid use, n (%)	4(8)
Fall-provoking drugs: Antihypertensives	34(68)
Vasodilators	9(18)
Antipsychotics	1(2)
Haemoglobin level, g/dL mean (SD)	9.29 (1.60)
Bone Markers	
Calcium, mg/dL, mean (SD)	9.68 (0.99)
Phosphate, mg/dL, mean (SD)	4.56 (2.30)
Parathyroid hormone (PTH), pg/mL, mean (SD)	211.74 (242.98)
Alkaline phosphatase (ALP), U/L, mean (SD)	201.04 (146.38)
Vitamin D, ng/mL, mean (SD)	6.21(4.79)
BMD T-Score (L1-L4), mean (SD)	−0.958 (1.57)
Total Hip T-Score, mean (SD)	−0.584 (1.19)
Upper Distal Radius T-Score, mean (SD)	−2.140 (1.29)

Values are given as mean (standard deviation) mean (SD) and as n (number) and (%) percentage; *BMI* body mass index, *FRAX* fracture risk assessment tool, *FRAT* fall risk assessment tool, *ADL* activities of daily living, *TUG* timed get up and go test, *MNA* mini nutritional assessment, *MMSE* mini mental state examination, *GDS−15* geriatric depression scale short form, *Kt/v* Kt/V is a number used to quantify hemodialysis treatment adequacy. K – dialyzer clearance of urea/t – dialysis time V – volume of distribution of urea, *BP* blood pressure, *BMD* bone mineral density

### Bone Mineral Density (BMD) Assessments

BMD assessments revealed a high prevalence of reduced bone mass among the study cohort. Overall, osteoporosis was identified in 52% of patients, while osteopenia was present in 42%; only 6% had normal BMD across all measured sites. Patients were categorized into three groups: osteoporosis, osteopenia, and normal based on WHO criteria and clinical thresholds. The findings are summarized in Supplementary Table 1.

Site-specific analysis showed that osteoporosis was most prevalent at the upper distal radius (46%), followed by the lumbar spine (20%) and the total hip (10%). Osteopenia was observed in 36% of patients at both the lumbar spine and radius, and in 24% at the total hip. Normal BMD was more frequently observed at the total hip (66%) compared to the lumbar spine (44%) and radius (18%).

### Falls and Fractures

Patients were further categorized as patients with “falls” and patients with “no falls" and those with VCF versus those without VCF. Table 2 shows the characteristics of falls and fractures of the study group. During the 6-month follow-up period, 28% of participants reported at least one new fall, most (16%), occurring on non-dialysis days. Of those who fell, 22% had a single fall and 6% had multiple, mainly due to imbalance.

**Table 2:** Prevalence and 6-Month Incidence of Falls and Fractures Among Study Participants (*n* = 50)

Prevalence	n (% of total cohort)	Time frame/unit
Past History of Falls	16 (32)	At baseline
Past History of Fractures	8 (16)	
Vertebral Compression Fractures (VCF)	7 (14)	
Non-VCF	1 (2)	
Incidence (New Events)		
New falls		6- month follow-up period
One fall	11 (22)	
Two falls	3 (6)	
New Vertebral Compression Fractures		
Single Fracture	1(2)	
Double Fractures	6 (12)	

Prevalence reflects conditions present at baseline. Incidence reflects new events occurring during the 6-month follow-up period

Most injuries were minor. Prior falls were statistically significantly more common among the falls group (78.6% vs. 13.9%, p < 0.001). Eight patients (16%) had a history of fractures; 14% of these were associated with VCF. Additionally, 14% were newly diagnosed with VCF during the study.

### Comprehensive Geriatric Assessment (CGA) Findings

Supplementary Table 2 highlights notable differences across the patients with falls/with no falls and patients with/without VCF. Patients with VCF showed worse functional and nutritional outcomes: lower ADL scores (5.14 vs. 5.35), slower TUG times (19.0 vs. 16.9 s), and poorer MNA scores (24.64 vs. 26.14). Depression was more pronounced (GDS-15: 9.43 vs. 8.1), and fracture risk was elevated (FRAX MOF score).

Patients with falls also exhibited greater impairments: lower ADL scores (4.57 vs. 5.61), slower TUG times (18.9 vs. 16.5 s), lower MNA scores (24.64 vs. 26.43), and higher depression scores (GDS-15: 9.57 vs. 7.78); all differences were statistically significant (p < 0.05). Frailty was higher among the patients with falls (4.43 vs. 3.56), though not statistically significant. Cognitive function (MMSE) did not differ meaningfully across groups.

### Comparison of Characteristics Between patients with Falls and patients with No Falls

Table 3 outlines significant differences between patients with falls and those with no falls. Patients with falls were older (69.5 vs. 67.2 years, *p* = 0.011) and had a higher prevalence of prior falls (78.6% vs. 13.9%, *p* < 0.001), reinforcing that age and fall history as key risk factors.

**Table 3:** Comparison of Characteristics Between Older HD Patients with Falls and with No Falls

Characteristic	Falls Group (*N* = 14)	No Falls Group (*N* = 36)	*p*-value
Age (years), mean (SD)	69.50 (3.29)	67.17 (3.01)	0.011 *
Body Mass Index (BMI, kg/m^2^), mean (SD)	26.5 (8.13)	30.0 (7.43)	0.059
Past Fall History (%)	78.6	13.9	< 0.001 *
Hemoglobin (Hb, g/dL), mean (SD)	8.54 (1.50)	9.58 (1.56)	0.040 *
Duration of Dialysis (years), mean (SD)	6.83 (5.04)	7.86 (4.69)	0.421
Ultrafiltration Volume, mean (SD)	2.57 (0.61)	3.0 (0.79)	0.075
Kt/V, mean (SD)	1.20 (0.26)	1.36 (0.30)	0.097
Charlson Comorbidity Index (CCI)	3.5 (2.56)	2.58 (1.38)	0.434
Activities of Daily Living Scale (ADL), mean (SD)	4.57 (2.17)	5.61 (1.15)	0.039 *
Geriatric Depression Scale 15 (GDS 15), mean (SD)	9.57 (1.27)	7.78 (2.31)	0.011 *
Timed "Get Up and Go" Test (TUG) in seconds, mean (SD)	18.86 (3.27)	16.53 (3.30)	0.037 *
Mini Mental Status Examination (MMSE), mean (SD)	26.0 (3.26)	27.03 (1.99)	0.281
Mini Nutritional Assessment (MNA), mean (SD)	24.64 (3.36)	26.43 (1.42)	0.032 *
Fall Risk Assessment Tool (FRAT), mean (SD)	8.14 (2.53)	4.83 (2.62)	< 0.001 *
Frailty Index, mean (SD) mean (SD)	4.43 (2.20)	3.56 (1.53)	0.253
Spinal T- Score (L1–L4), mean (SD)	−1.02 (1.53)	−0.933 (1.60)	0.837
Total Hip T- Score, mean (SD)	−0.936 (0.909)	−0.447 (1.27)	0.122
Upper Distal 1/3 Radius T- Score, mean (SD)	−2.48 (1.05)	−2.0 (1.36)	0.266
Calcium (Ca, mg/dL), mean (SD)	9.50 (0.70)	9.75 (1.08)	0.626
Phosphorus (Ph, mg/dL), mean (SD)	4.08 (2.07)	4.75 (2.39)	0.289
Parathyroid Hormone (PTH, pg/mL), mean (SD)	137.18 (111.7)	240.7 (273.7)	0.310
Alkaline Phosphatase (ALP, U/L), mean (SD)	221.0 (169.1)	193.1 (138.3)	0.795
25-hydroxy Vitamin D (Vit D, ng/mL), mean (SD)	5.12 (3.56)	6.63 (5.17)	0.627
Vision Problems (%)	78.6	50.0	0.110
Hearing Problems (%)	14.3	19.4	0.717
Chronic Liver Disease (%)	28.6	5.6	0.044 *
Hypertension (%)	64.3	69.4	0.746
Diabetes Mellitus (%)	35.7	13.9	0.118

Values are given as mean (standard deviation) mean (SD) and as n(number) and (%) percentage; *BMI* body mass index, *FRAX* fracture risk assessment tool, *ADL* activities of daily living, *TUG* timed get up and go test, *MNA* mini nutritional assessment, *MMSE* mini mental state examination, *GDS−15* geriatric depression scale short form, *Kt/v* Kt/V is a number used to quantify hemodialysis treatment adequacy. K – dialyzer clearance of urea/t – dialysis time V – volume of distribution of urea, *BMD* bone mineral density, p*: p value of Mann Whitney test, significant at level <0.05*, *n* = number, *%* = percentage

Anemia was more pronounced in patients with falls (hemoglobin: 8.5 vs. 9.6 g/dL, *p* = 0.040), contributing to impaired balance. Functional deficits were evident with lower ADL scores, slower TUG times, and greater nutritional decline. Depression (higher GDS-15 scores) and chronic liver disease (28.6% vs. 5.6%, *p* = 0.044) were also more frequent among patients with falls. Heightened fall risk was confirmed by the statistically significantly elevated FRAT scores (8.1 vs. 4.8, *p* < 0.001).

### Comparison of Characteristics Between VCF and Non-VCF Patients

Table 4 also depicts key differences between patients with and without VCF. An earlier menopause age was statistically significantly associated with VCF (45.5 vs. 47.7 years, *p* = 0.049), reflecting increased bone fragility due to reduced estrogen. Back pain was markedly more common among VCF patients (85.7% vs. 39.5%, *p* < 0.05).

**Table 4:** Comparison of Characteristics Between Older HD Patients with VCF and without VCF

Characteristic	Non-VCF	VCF	*p*-value
Age (years), mean (SD)	67.81(3.24)	67.86 (3.43)	0.848
Age of menopause (years), mean (SD)	47.67 (1.87)	45.45 (0.50)	0.049
BMI kg/m^2^, mean (SD)	28.71 (7.30)	30.90 (0.38)	0.622
Duration of Dialysis (years), mean (SD)	7.48 (4.70)	8.14 (5.45)	0.956
Kt/V mean (SD)	1.3 (0.30)	1.4 (0.25)	0.350
Back Pain (%)	39.5%	85.7%	< 0.05
Charlson Comorbidity Index, mean (SD)	2.98 (1.80)	2.0 (1.0)	0.157
FRAX Hip Score mean (SD)	0.006 (0.004)	0.01 (0.008)	0.303
FRAX MOF Score mean (SD)	0.057 (0.016)	0.073 (0.003)	< 0.05
Spinal T Score (L1–L4), mean (SD)	−0.921 (1.58)	−1.18 (1.57)	0.681
Total Hip T Score, mean (SD)	−0.391(1.11)	−1.77 (1.05)	0.003
Upper Distal Radius T Score, mean (SD)	−2.00 (1.30)	−2.95 (0.92)	0.056
ADL, mean (SD)	5.35 (1.57)	5.14 (1.57)	0.681
TUG Test (s), mean (SD)	16.88 (3.56)	19.0 (1.73)	0.126
MMSE, mean (SD)	26.72 (2.42)	27.0	0.642
MNA, mean (SD)	26.14 (2.15)	24.64	0.119
GDS-15, mean (SD)	8.10 (2.30)	9.43 (1.27)	0.166
Frailty Index, mean (SD)	3.7 (1.8)	4.0 (1.0)	0.565
Calcium (Ca), mg/dl, mean (SD)	9.71(1.03)	9.48 (0.71)	0.743
Phosphate (Ph), mg/dl, mean (SD)	4.38 (2.18)	5.68 (2.89)	0.270
Parathyroid Hormone (PTH), mg/dl, mean (SD)	200.1(255.3)	283.2 (138.0)	0.052
Alkaline Phosphatase (ALP), IU/L, mean (SD)	166.3 (99.9)	414.0 (207.8)	0.003
Vitamin D (Vit D), ng/ml, mean (SD)	6.53 (4.83)	4.28 (4.37)	0.224

Values are given as mean (standard deviation) mean (SD) and as n(number) and (%) percentage; *BMI* body mass index, *FRAX* fracture risk assessment tool, *ADL* activities of daily living, *TUG* timed get up and go test, *MNA* mini nutritional assessment, *MMSE* mini mental state examination, *GDS−15* geriatric depression scale short form, *Kt/v* Kt/V is a number used to quantify hemodialysis treatment adequacy. *K* – dialyzer clearance of urea/t – dialysis time *V* – volume of distribution of urea, *BMD* bone mineral density, p*: *p* value of Mann Whitney test, significant at level <0.05*, *n* = number; *%* = percentage

Fracture risk was elevated in this group, as shown by higher FRAX MOF scores (0.073 vs. 0.057, *p* < 0.05). Although functional performance (ADL, TUG), nutritional status (MNA), and depression scores (GDS-15) were worse in the VCF group, these differences were not statistically significant.

### Biochemical and Bone Marker Correlations

Statistically significant relationships between biochemical and bone health indicators in older patients on HD are shown in Supplementary Table 3. Bone specific ALP showed strong inverse correlations with total hip T-scores (*p* = 0.003) and radius T-scores (*p* = 0.008), indicating reduced bone density with elevated ALP levels. FRAX hip scores were also negatively correlated with total hip (*p* = 0.032) and radius T-scores (*p* = 0.019), reflecting higher fracture risk in those with lower BMD Supplementary Table 3.

## DISCUSSION

Chronic Kidney Disease (CKD) is a prevalent growing global health challenge, particularly among older populations with severe End-Stage Renal Disease (ESRD) affecting approximately 7% of the population^1,3,6^.

This study highlights the complex interplay of clinical, functional, and biochemical factors contributing to falls, fractures, and compromised bone health in older patients on HD. Incorporating geriatric assessments and comparative data enhances insight into these vulnerabilities. The cohort’s demographic and clinical profile further classifies them as a high-risk group prone to adverse outcomes.

Falls affected 28% of patients and were primarily associated with vision impairment, anemia, dizziness (often hypoglycemia-related), hypotension, and environmental hazards. Prior falls strongly predicted future events, and 12% resulted in fractures which emphasizes the importance of early fall prevention strategies^19,20^. This study aligns with previous research on fall prevalence in older patients on HD, with notable findings that complement the literature. The fall rate observed (28%) is comparable to Sai A et al.'s report of a 24.4% fall rate but lower than van Loon et al.'s 47% over two years^21,22^. While literature often reports increased fall risk in postmenopausal women due to bone density loss, no significant gender difference emerged in our cohort^23^.

Anemia emerged as a significant risk factor, this is consistent with findings by Hopstock et al. emphasizing how anemia-induced fatigue, dizziness, and muscle weakness exacerbate fall risk in older patients^24^. Smoking (previously associated with increased fall risk in older men), BMI (previous research suggesting that obesity might offer some protection against severe fall-related injuries, and most comorbidities except for chronic liver disease, did not show significant associations^24–27^. Despite high comorbidity (CCI 2.84), no significant correlation with falls was observed, possibly due to enhanced care.

Dialysis-related factors such as session duration, Kt/V, and intradialytic blood pressure changes were not significantly associated with falls. Likewise, polypharmacy and medications known to provoke falls, lacked statistical significance. These findings are consistent with those reported by Polinder-Bos et al. and Zhou et al.^28,29^.

Fall risk in this population is likely multifactorial, arising from the interplay of physiological vulnerabilities, dialysis-related factors, medication effects, environmental hazards, and psychosocial determinants.

Functional and psychological assessments (e.g., TUG, GDS-15, ADL, MNA, and FRAT) were consistently poorer among patients with falls, highlighting vulnerabilities stemming from mobility deficits, malnutrition, and depression. These deficits echoed prior findings and reinforced the need for comprehensive, interdisciplinary management^30–33^.

Frailty and BMD conventionally associated with falls were not independently linked in our cohort, likely due to their high baseline prevalence masking their contribution. The prevalence of low BMD and frailty in this cohort may have diluted its statistical impact, suggesting the need for further exploration of subtler bone-health predictors of falls^34^.

### Bone Mineral Density and Fracture Risk

BMD assessments revealed substantial skeletal fragility, with osteoporosis most common at the upper distal radius (46%), lumbar spine (20%), and hip (10%). Osteopenia was prevalent, emphasizing the need for site-specific interventions to strengthen bone health and reduce fracture risk. Biochemical markers showed statistically significant correlations with BMD scores: elevated ALP levels correlated with reduced T-scores at the hip and distal radius, aligning with the findings of Chen et al.^35^. Both PTH and ALP emerged as key predictors of bone quality and fracture risk, supporting their role as therapeutic targets and aligning with the findings reported by Matias et al.^36^. The inverse relationship between FRAX hip scores and BMD further illustrates the complexities of fracture prediction in dialysis patients. No significant association was found between phosphate levels and BMD, aligning with Sit et al. but contrasting with Genest et al. possibly reflecting differences in phosphate management^37,38^. Similarly, vitamin D levels did not correlate with BMD, potentially due to widespread deficiency in the cohort or overriding effects of ALP and PTH^39^.

### Vertebral Compression Fractures (VCF)

VCF were observed in 14% of patients and were associated with increased back pain, poorer functional performance and skeletal fragility. Early menopause was statistically significantly associated with VCF, likely through oestrogen-related bone loss. Reduced estrogen likely contributes to bone fragility in this group^40^. Our findings align with those of Tomé-Bermejo et al. that highlight the role of hormonal and mechanical factors in VCF etiology^41^. VCF patients had higher FRAX-MOF scores, elevated ALP, lower vitamin D, and reduced hip BMD highlighting their elevated fracture risk^42^. However, as observed in Babayev and Jirasirirak’s studies, even though FRAX is widely used in clinical practice, it may not accurately capture fracture risk in CKD patients as it omits renal-specific risk factors, underscoring the need for adapted prediction models^43,44^.

Functional impairments including lower ADL scores and prolonged TUG times further distinguished the VCF group, emphasizing the impact of vertebral fragility on independence and quality of life.

### Falls and Fractures Relationship

The observed link between falls and fractures in this study suggests that recurrent falls and their consequences may contribute to fragility fractures, consistent with findings by Kaneko et al.^45^.

Research indicates that impaired mobility and balance issues increase fracture risk. Additionally, some studies suggest that imbalances in calcium-phosphorus metabolism may be associated with fracture risks in dialysis patients, though the specific impact requires further exploration^34,35^.

### Strengths and Limitations

This study provides detailed insight into the risk factors for falls, bone loss, and VCF in older HD patients. Key contributors include anemia, hypotension, and gender-specific hormonal influences. By assessing fall timing and underlying causes, we offer practical considerations for individualized interventions. The study also acknowledges that existing tools like FRAX may not fully account for CKD-specific fracture risk factors, suggesting a need for further refinement of prediction models. While FRAX is widely used to estimate fracture risk, it does not account for several factors highly relevant to older patients on HD such as dialysis-related hypotension, intradialytic falls, frailty, cognitive impairment, nutritional status, and bone turnover markers consequently limiting its predictive accuracy in this population. The aforementioned factors should thus be taken into account in prediction models.

Additionally, proactive strategies such as regular BMD evaluations and geriatric assessments including measures like ADLs, TUG, MNA, MMSE, and GDS-15 may help identify patients who could benefit from early interventions aimed at reducing falls, fractures, and functional decline.

However, several limitations apply: the small cohort size limits generalizability; the cross-sectional design prevents causal inference; reliance on self-reported falls introduces recall bias; and absence of long-term follow-up hinders outcome tracking. Variability in vitamin D and phosphate-related findings highlights the importance of population-specific protocols.

Future research should aim to include larger, more diverse samples and use longitudinal, objectively measured fall data to validate and extend these findings.

### Conclusion and Implications for Clinical Practice

Our findings underscore the importance of adopting a comprehensive, personalized, and multidisciplinary care approach for older patients on HD who are at risk of falls and fractures. Functional impairments, psychological vulnerability and environmental hazards contribute to fall risk and must be addressed through proactive screening and intervention strategies.

Bone health management should extend beyond conventional protocols, prioritizing correction of PTH and ALP abnormalities, vitamin D repletion, and site-specific skeletal assessment. The routine use of geriatric evaluation tools such as the TUG test and ADL assessment are valuable in stratifying fall and fracture risk and guiding timely interventions.

Furthermore, the limited predictive accuracy of FRAX in CKD populations highlights the need for renal-specific fracture risk models that integrate unique pathophysiologic factors associated with CKD and dialysis. Longitudinal studies are warranted to evaluate intervention efficacy and enhance fracture prediction in this vulnerable group.

## Supplementary Information

Below is the link to the electronic supplementary material.

Supplementary file1 (DOCX 23 KB)

## Data Availability

Not applicable.
