# Supplementary material for: Association of Bone Mass, Falls, and Vertebral Fractures in Older Patients on Hemodialysis: The Role of Comprehensive Geriatric Assessment
Source: J Gen Intern Med. 2026 May 6;41(10):2835–43. doi: 10.1007/s11606-026-10398-3 (PMC13421600; doi:10.1007/s11606-026-10398-3)
Supplement: Supplementary file 1 — Supplementary file1 (DOCX 23 KB) [file 11606_2026_10398_MOESM1_ESM.docx]

**Supplementary Table 1.** **Bone Mineral Density Measurements**

| **Bone Site** | **Mean T-score (SD)** | **Normal**  **(T score> -1.0)** | **Osteopenia**  **(T score -2.5 to -1.0)** | **Osteoporosis**  **(T score ≤ -2.5)** |
| --- | --- | --- | --- | --- |
| **Total Hip** | -0.584 (1.19) | 66% (n= 33) | 24% (n=12) | 10% (n=5) |
| **Lumbar Spine**  **(L1-L4)** | -0.958 (1.57) | 44% (n=22) | 36% (n=18) | 20% (n=10) |
| **Upper distal 1/3 of radius** | -2.140 (1.29) | 18% (n=9) | 36% (n=18) | 46% (n=23) |

^n = number; % = percentage; L = lumbar^

|  |  |  |  | **Supplementary Table 2. Comprehensive Geriatric Assessment (CGA) Findings: Group Comparisons**   \| **Assessment** \| **Mean (SD)** \| **Non-VCF** \| **VCF** \| **Falls** \| **No Falls** \| \| --- \| --- \| --- \| --- \| --- \| --- \| \| **Activities of Daily Living (ADLs)** \| 5.32 (1.55) \| 5.35 (1.57) \| 5.14 (1.57) ** \| 5.61 (1.15) \| 4.57 (2.17) * \| \| **Timed "Get Up and Go" (TUG)** \| 17.18 (3.43) \| 16.88 (3.56) \| 19.0 (1.73) ** \| 16.53 (3.30) \| 18.86 (3.27) * \| \| **Mini-Mental State Exam (MMSE)** \| 26.76 (2.42) \| 26.72 (2.42) \| 27.0 (2.58) \| 27.03 (1.99) \| 26.0 (3.26) # \| \| **Mini Nutritional Assessment (MNA)** \| 25.93 (2.26) \| 26.14 (2.15) \| 24.64 (2.67) ** \| 26.43 (1.42) \| 24.64 (3.36) * \| \| **Geriatric Depression Scale (GDS-15)** \| 8.28 (2.24) \| 8.1 (2.3) \| 9.43 (1.27) ** \| 7.78 (2.31) \| 9.57 (1.27) * \| \| **Frailty Index** \| 3.80 (1.77) \| 3.7 (1.8) \| 4.0 (1.0) ** \| 3.56 (1.53) \| 4.43 (2.20) # \| \| **FRAX Hip Score** \| 0.006 (0.005) \| 0.006 (0.004) \| 0.01 (0.008) ** \| N/A \| N/A \| \| **FRAX MOF Score** \| 0.059 (0.016) \| 0.057 (0.016) \| 0.073 (0.003) * \| N/A \| N/A \|   *** ^significant difference in the falls group, p< 0.05; # non-significant difference, p>0.05,^ ** ^significant difference in VCF group; VCF = vertebral compression fractures, FRAX = fracture risk assessment tool^** |  |  |  |  |  |  |
| --- | --- | --- | --- | --- | --- | --- | --- | --- | --- | --- | --- | --- | --- | --- | --- | --- | --- | --- | --- | --- | --- | --- | --- | --- | --- | --- | --- | --- | --- | --- | --- | --- | --- | --- | --- | --- | --- | --- | --- | --- | --- | --- | --- | --- | --- | --- | --- | --- | --- | --- | --- | --- | --- | --- | --- | --- | --- | --- | --- | --- | --- | --- | --- | --- |

**Supplementary Table 3. Correlation Between Biochemical Markers, Bone Mineral Density (BMD) Scores, and FRAX Scores in Older Patients on Hemodialysis**

| **Calcium (mg/dl)** | **Rs** | | **P** | |
| --- | --- | --- | --- | --- |
| Phosphorous | 0.329 | | 0.020* | |
| Alkaline phosphatase | 0.059 | | 0.686 | |
| 25-hydroxy vitamin D | -0.011 | | 0.939 | |
| Parathyroid hormone | -0.318 | | 0.024* | |
| **Phosphorous (mg/dl)** |  | |  | |
| Alkaline phosphatase | 0.059 | | 0.686 | |
| 25-hydroxy vitamin D | 0.021 | | 0.884 | |
| Parathyroid hormone | 0.121 | | 0.401 | |
| **Alkaline phosphatase (IU/L)** |  | |  | |
| 25-hydroxy vitamin D | -0.206 | | 0.152 | |
| Parathyroid hormone | 0.335 | | 0.018* | |
| **25-hydroxy vitamin D (ng/ml)** |  | |  | |
| Parathyroid hormone | -0.137 | | 0.344 | |
| **Calcium (mg/dl)** | | **R** | **P** |  |
| T score spines L1-L4 | | -0.089 | 0.541 |  |
| Total hip T score | | 0.055 | 0.703 |  |
| Upper Distal 1/3 of radius T score | | 0.043 | 0.766 |  |
| **Phosphorous (mg/dl)** | |  |  |  |
| T score spines L1-L4 | | -0.140 | 0.334 |  |
| Total hip T score | | 0.151 | 0.249 |  |
| Upper Distal 1/3 of radius T score | | 0.146 | 0.313 |  |
| **Alkaline phosphatase (IU/L)** | |  |  |  |
| T score spines L1-L4 | | -0.064 | 0.657 |  |
| Total hip T score | | -0.406 | 0.003* |  |
| Upper Distal 1/3 of radius T score | | -0.371 | 0.008* |  |
| **25-hydroxy Vitamin D (ng/ml)** | |  |  |  |
| T score spines L1-L4 | | 0.040 | 0.784 |  |
| Total hip T score | | -0.005 | 0.970 |  |
| Upper Distal 1/3 of radius T score | | 0.055 | 0.703 |  |
| **Parathyroid hormone (Pg/ml)** | |  |  |  |
| T score spines L1-L4 | | 0.057 | 0.694 |  |
| Total hip T score | | 0.062 | 0.670 |  |
| Upper Distal 1/3 of radius T score | | 0.148 | 0.305 |  |
| **FRAX hip score** | **R** | | **P** |  |
| T score spines L1-L4 | 0.058 | | 0.813 |  |
| Total hip T score | -0.492 | | 0.032* |  |
| Upper Distal 1/3 of radius T score | -0.531 | | 0.019* |  |
| **FRAX MOF score** |  | |  |  |
| T score spines L1-L4 | 0.119 | | 0.627 |  |
| Total hip T score | -0.375 | | 0.113 |  |
| Upper Distal 1/3 of radius T score | -0.398 | | 0.091 |  |

**^FRAX = fracture risk assessment tool; MOF = major osteoporotic fracture^**

**Legend of Tables**

- - - 1. **Supplementary Table 1** shows the bone mineral density measurements of the study population.
      2. **Supplementary Table 2** shows the comprehensive geriatric assessment (CGA) findings in both the falls and no-falls group and in the VCF and non-VCF HD patients.
      3. **2 Supplementary Table 3** depicts the correlation between biochemical markers, bone mineral density (BMD) scores, and FRAX scores in older patients on HD.
